# The Antioxidant Peroxiredoxin 6 (Prdx6) Exhibits Different Profiles in the Livers of Seawater- and Fresh Water-Acclimated Milkfish, *Chanos chanos*, upon Hypothermal Challenge

**DOI:** 10.3389/fphys.2016.00580

**Published:** 2016-11-29

**Authors:** Chia-Hao Chang, Wan-Yu Lo, Tsung-Han Lee

**Affiliations:** ^1^Department of Life Sciences, National Chung Hsing UniversityTaichung, Taiwan; ^2^Department of Biotechnology, Hung Kuang UniversityTaichung, Taiwan; ^3^Agricultural Biotechnology Center, National Chung Hsing UniversityTaichung, Taiwan

**Keywords:** milkfish, liver, peroxiredoxin 6, oxidative stress, low temperature, seawater, fresh water

## Abstract

A tropical species, the euryhaline milkfish (*Chanos chanos*), is a crucial economic species in Southeast Asia and is intolerant of water temperature below 12°C. Large numbers of milkfish die during cold periods in winter. Hypothermal environments usually increase oxidative stress in teleosts, and the liver is the major organ for anti-oxidative responses in the body. Peroxiredoxin-6 (Prdx6) in mammals is a multi-functional enzyme and acts as both glutathione peroxidase, phospholipase A_2_ and acyl-transferase for maintenance of redox status and prevention of cell membrane peroxidation. Prdx6 can protect cells from oxidant-induced membrane damage by translocating the Prdx6 protein from the cytosol to the membrane. Upon cold stress, *Ccprdx6* transcript levels were up-regulated after 24 h and 96 h in livers of fresh water (FW)- and seawater (SW)-acclimated milkfish, respectively. In the hypothermal FW group, the Prdx6 protein was up-regulated in the cytosol of hepatocytes with a similar role as glutathione peroxidase to reduce oxidative stress upon hypothermal challenge. Conversely, in hypothermal SW milkfish, total Prdx6 protein was down-regulated. However, cytosolic Prdx6 protein was translocated to the membrane, using the ability of phospholipase A_2_ to stabilize the membrane redox state. Moreover, H_2_O_2_ content was increased in FW-acclimated milkfish livers upon hypothermal challenge. *Ex vivo* H_2_O_2_ treatment of milkfish livers also induced *Ccprdx6* transcriptional expression, which provided more evidence of the antioxidant role of milkfish Prdx6. Taken together, upon hypothermal challenge, greater oxidative stress in livers of FW-acclimated milkfish rather than SW-acclimated individuals led to different profiles of hepatic CcPrdx6 expression between the FW and SW group. The results indicated that CcPrdx6 played the role of antioxidant with different mechanisms, i.e., binding to reactive oxygen species and stabilizing membrane fluidity, in livers of hypothermal FW and SW milkfish, respectively.

## Introduction

Environmental temperature is an important factor affecting many physiological and biochemical processes in ectotherms. Most fishes are ectotherms with the best performance of individuals (e.g., growth, exercise, reproduction) in the optimal temperature range. Once out of the optimal range, fluctuating environmental temperatures increase the formation rate of reactive oxygen species (ROS) (Abele and Puntarulo, 2004; Heise et al., 2006; Pörtner et al., 2007; Tseng et al., 2011). The ROS consist of the superoxide anion (${\text{O}}_{2}^{-}$), hydrogen peroxide (H_2_O_2_), and hydroxyl radicals (OH^−^). Enhanced production of ROS can be reactive to cellular components, such as oxidation of proteins, peroxidation of lipids, damage to DNA strands, and inhibition of enzyme activity, and can eventually lead to cell death (Hermes-Lima and Zenteno-Savin, 2002). However, antioxidant enzymes play an important role in the neutralization of ROS and reduce oxidative stress that causes damage to cellular components. Superoxide dismutase (SOD), glutathione peroxidase (GPx), catalase (CAT), and peroxiredoxin (Prdx) are major antioxidant enzymes for detoxification of different kinds of ROS and protection of cells against oxidative stress (Hermes-Lima and Zenteno-Savin, 2002; Wang et al., 2008; Fisher, 2011).

The Prdx protein family contains six members of thiol-specific antioxidant proteins in mammals and teleosts and is categorized into three groups: typical 2-Cys (Prdx 1–4), atypical 2-Cys (Prdx5), and 1-Cys (Prdx6) (Wood et al., 2003; Pérez-Sánchez et al., 2011). Among them, the typical 2-Cys Prdx group has two conserved catalytic cysteine residues at the N- and C-terminus. Prdx1 and Prdx2 are located in the cytosol, and are also called the natural killer enhance factors (NKEF-A, NKEF-B) (Dong et al., 2007; Pérez-Sánchez et al., 2011), whereas Prdx3 is located in the mitochondria (Choi et al., 2014), and Prdx4 is located in the endoplasm reticulum and extracellular space (Konno et al., 2015). Located in the cytosol, the atypical 2-Cys group, the Prdx5, has one conserved cysteine and another non-conserved cysteine for the catalytic cycle (Choi et al., 2016). Prdx6 is a thioredoxin-like protein that has a single active-site Cys-residue with the ability to bind and reduce phospholipid hydroperoxides. Unlike the other Prdx protein members, Prdx6 has dual functions, including glutathione peroxidase and phospholipase A_2_ activities (Fisher, 2011). In addition, this protein has two conserved enzyme active sites: the peroxidase motif is PVCTTE containing a 1-Cys active site at the N-terminus catalytic center, and the lipase motif has GXSXG at position 30–34 of which the Ser^32^ and His^26^ are crucial for PLA_2_ activity of Prdx6 (Manevich et al., 2007; Fisher, 2011; Priyathilaka et al., 2016).

Expression of Prdx6 has been reported in teleosts with different treatments, including live bacteria (Yu et al., 2009; Zheng et al., 2010), virus (De Zoysa et al., 2012), pathogen-associated molecular patterns (PAMP; Zheng et al., 2010; Pérez-Sánchez et al., 2011; De Zoysa et al., 2012; Mu et al., 2013; Priyathilaka et al., 2016), H_2_O_2_ (Zheng et al., 2010; Priyathilaka et al., 2016), and heat stress (Tolomeo et al., 2016). Among these studies, the antioxidant and protective functions of recombinant Prdx6 under oxidative stress were assayed (Zheng et al., 2010; Priyathilaka et al., 2016). With increasing temperature, the Antarctic emerald rockcod (*Trematomus bernacchii*) demonstrated up-regulation of *prdx6b* and down-regulation of *prdx6a* mRNA abundance (Tolomeo et al., 2016). Moreover, *prdx5* and *prdx6* were found in thermally stressed Antarctic bivalves (*Laternula elliptica*) during ESTs analysis, and *prdx5* and *prdx6* mRNA expression were elevated in the gill and digestive glands with time-course heat treatment (Park et al., 2008). In mammalian studies, Prdx6 was reported to be an antioxidant enzyme that protected the lung epithelial cells from H_2_O_2_-induced oxidative stress (Wang et al., 2008). On the other hand, Prdx6 has its maximal activity at an acidic pH (oxidative status) and thus reduces phospholipid peroxidation to neutral status in repaired cell membranes (Manevich et al., 2009, 2013). Under heat stress, Prdx6 was translocated to the membrane of erythrocytes to stabilize membrane fluidity and reduce damage from thermal stress (Sharma et al., 2013).

The milkfish (*Chanos chanos*) is an economically important species that is widely farmed in Southeast Asia. Being a marine euryhaline teleost inhabiting the tropical and subtropical zone of the Indo-Pacific Ocean (Bagarinao, 1994), milkfish are intolerant to temperatures lower than 12°C (Kang et al., 2015). Moreover, SW (seawater)-acclimated milkfish exhibited higher hypothermal tolerance than FW (fresh water)-acclimated milkfish to critical thermal minimum (CT_Min_) analysis (Kang et al., 2015). Hsieh and Kuo (2005) reported that under cold stress (15°C) FW-acclimated milkfish would regulate desaturation of fatty acids to maintain membrane fluidity. Under non-lethal low temperatures (18°C), gill Na^+^, K^+^-ATPase (NKA) activity of SW milkfish showed a partial compensatory response to moderate cold effects and maintained ion homeostasis. Furthermore, relative amounts of heat shock protein 70 in milkfish gills were increased to stabilize protein structure under temperature fluctuations (Kang et al., 2015). The proteomic analyses of milkfish livers under hypothermal stress detected a pI shift of two Prdx6 protein spots (Chang et al., 2016). From the transcriptome database of milkfish with low-temperature treatments (Hu et al., 2015), the partial sequence of *Ccprdx6* could be identified.

In this study, the full-length *Ccprdx6* cDNA sequence was further identified. The abundance of *CcPrdx6* mRNA and protein in FW and SW-acclimated milkfish livers under hypothermal stress was determined. The membrane fraction of milkfish livers was analyzed by immunoblotting to reveal whether the translocation of CcPrdx6 protein occurred upon hypothermal challenge. H_2_O_2_content, as an indicator of ROS levels, was measured in livers of hypothermal FW and SW milkfish to compare the salinity effects on oxidative stress caused by low temperatures. In addition, *ex vivo* experiments of H_2_O_2_ treatment were performed to demonstrate that *Ccprdx6* mRNA was expressed in response to oxidative stress. Finally, hepatic Prdx6 expression profiles between SW- and FW-acclimated milkfish upon hypothermal challenge were compared to elucidate the differences in potential mechanisms for the roles of an antioxidant.

## Materials and methods

### Experimental animals

The juvenile milkfish (average total length: 9.3 ± 0.1 cm; average body weight: 10.4 ± 0.5 g) were purchased from a local fish farm in Taiwan. Experimental animals were maintained in seawater (SW; 35‰) and fresh water (FW) at 28 ± 1°C with a 12/12 h light/dark photoperiod for at least 1 month. The water was continuously circulated through fabric-floss filters, and fish were fed daily with commercial pellets. The water of hypothermal SW and FW groups was cooled at a constant rate (2°C h^−1^) from 28° to 18°C with cooling systems (PF-225M, PRINCE, Tainan, Taiwan). After the temperature reached 18°C, the experimental groups of milkfish were sampled at 1, 3, 6, 12, 24, 48, 96, and 168 h (1 week). For the following analyses, the control groups were kept in SW or FW at 28°C before sampling. The fish were fed commercial pellets daily, but were not fed for 24 h before sampling. The protocol for the experimental fish was reviewed and approved by the Institutional Animal Care and Use Committee (IACUC) of the National Chung Hsing University (IACUC Approval No. 105-024 to THL).

### Total RNA extraction and reverse transcription

Total RNA samples were isolated from livers of milkfish using the TriPure Isolation Reagent (Roche, Mannheim, Germany) following the manufacturer's instructions. The RNA pellet was dissolved in 50 μL DEPC-H_2_O using the RNAspin Mini RNA isolation kit (GE Health Care, Piscataway, NJ, USA) to eliminate genomic DNA contamination according to the manufacturer's instructions. The quality of the extracted RNA was determined by (i) the A260/A280 ratio (2.0–2.2) using the NanoDrop 2000 (Thermo, Wilmington, CA, USA); (ii) RNA electrophoresis. Total RNA concentrations of all samples measured by the NanoDrop 2000 (Thremo) were 0.3–0.5 μg μL^−1^. First-strand cDNA was synthesized by reverse transcribing 1 μg of the total RNA using the iScript Reverse Transcription Supermix (Bio-Rad Laboratories, Hercules, CA, USA), following the manufacturer's instructions.

### Identification of *Ccprdx6* cDNA sequence

Partial DNA sequence presenting homology to *Ccprdx6* was identified from the milkfish NGS database (Hu et al., 2015). The partial fragment of the *Ccprdx6* gene was amplified by PCR using cDNA as a template, and the primers were designed by Primer 3 Plus (http://www.bioinformatics.nl/cgi-bin/primer3plus/primer3plus.cgi) based on the highly conserved region compared with that of other teleosts (Supplementary Table 1). The templates of cDNA for gene cloning were obtained using the SMART RACE cDNA amplification kit (Clontech, Palo Alto, CA, USA) following the manufacturer protocol. For PCR amplification, 2 μL cDNA was used as the template in a 50 μL reaction containing 0.25 μM dNTPs, 2 U Ex-Taq polymerase (Takara, Shiga, Japan), and 0.1 μM of each primer. PCR products were subcloned into the pGM-T vector (GeneMark, Taipei, Taiwan) and sequenced. The open reading frame of *Ccprdx6* was predicted with ORF finder (http://www.ncbi.nlm.nih.gov/gorf/gorf.html). EMBOSS Needle online server (http://www.ebi.ac.uk/Tools/psa/emboss_needle/) was applied to pairwise sequence alignment. The protein molecular weight and theoretical isoelectric point was predicated with ExPASy compute pI/Mw tool (http://web.expasy.org/compute_pi). Amino acid sequences for Prdx6 were aligned by ClustalW and a phylogenetic tree was constructed using MEGA 6. The tree was built using the neighbor-joining method with the pairwise deletion gaps calculation option.

### Quantitative real-time PCR

The *Ccprdx6* mRNA of livers was detected by KAPA SYBR FAST qPCR Kit Master Mix (Kapa Biosystems, Boston, MA, USA) and quantified with the Mini Opticon real-time PCR system (Bio-Rad). All qPCR primers were checked by (i) primer efficiency 90–105%, (ii) melting curve analysis, and (iii) presence of a single amplification product in a 1.5% agarose gel. The *Ccprdx6* mRNA values were normalized with the expression of *Ccgapdh* mRNA from the same cDNA samples. PCR reactions contained 8 μL of cDNA (100x dilution), 2 μL of qPCR primers (2 μM), and 10 μL of SYBR Master Mix (Kapa). One liver sample from SW-acclimated milkfish was used as the internal control among different groups. Relative gene expression was analyzed by the comparative Ct method with the formula 2^−^[(Ct_*Ccprdx*6,n_ − Ct_*Ccgapdh*,n_) − (Ct_Ccprdx6,c_ − Ct_Ccgapdh,c_)], where Ct corresponded to the threshold cycle number. For tissue distribution of *Ccprdx6* expression, the same internal control was used for normalization.

### Protein extraction and western immunoblotting

According to the method of Chang et al. (2016), milkfish livers were suspended in SEID medium (150 mM sucrose, 10 mM EDTA, 50 mM imidazole, 0.1% sodium deoxycholate, pH 7.5) containing protease inhibitor (vol/vol: 25:1; Roche) and homogenized by a Polytron PT1200E homogenizer (Lucerne, Switzerland) at maximal speed. The homogenates were then centrifuged at 10,000 × *g*, 4°C for 10 min. Protein concentrations of the supernatants were determined using reagents from the Protein Assay Kit (Bio-Rad), and bovine serum albumin (Sigma-Aldrich, St. Louis, MO, USA) was used as a standard. Aliquots containing 100 μg of homogenates were heated at 60°C for 15 min and fractionated by electrophoresis on SDS containing 10% polyacrylamide gels. The pre-stained protein molecular weight marker (#26616, Thermo) was applied in electrophoresis. The separated proteins were transferred to 0.45 μm PVDF blotting membranes (Millipore, Bedford, MA, USA). Then, the membranes were pre-incubated for 1 h in PBST with 5% (wt/vol) nonfat dried milk to minimize non-specific binding. The blots were incubated with the primary antibody (Prdx6, 1:3000, GTX115262, Genetex, Irvine, CA, USA; GAPDH, 1:5000, GTX100118, Genetex) overnight at 4°C, followed by incubation with the HRP-conjugated secondary antibody (goat anti-rabbit IgG, 1:10,000, GTX213110, Genetex) for 1 h at 28°C. The blots were developed with the Immobilon Western Chemiluminescent HRP substrate (Millipore). The images were photographed using the universal hood with a cooling-charge-coupled device (CCD) camera (ChemiDoc XRS^+^, Bio-Rad) and analyzed with the Image Lab software version 3.0 (Bio-Rad) to normalize numerical values compared with relative intensities of immunoreactive bands. The liver homogenates from SW-acclimated milkfish were used as the internal control among different blots. The intensity of the immunoreactive band of the internal control in the immunoblot was set to one and used as a standard for normalizing relative intensities of other bands in the immunoblot. Relative abundance of target proteins was calculated using the following formula (Prdx6_n_/GAPDH_n_)/(Prdx6_internal control_/GAPDH_internal control_).

### Preparation of liver membrane fractions

Milkfish liver samples in the homogenization buffer (250 mmol l^−1^ sucrose, 1 mmol l^−1^ EDTA, 30 mmol l^−1^ Tris base, pH = 7.6) containing protease inhibitor (vol/vol: 25:1; Roche) were homogenized using a Polytron PT1200E (Lucerne, Switzerland) homogenizer at maximal speed. The tissue debris, nuclei, and lysosomes were removed by low-speed centrifugation (1300 × *g* for 10 min, 4°C). The remaining supernatant was centrifuged at 20,800 × *g* for 1 h at 4°C. The pellet containing large amounts of membrane was suspended in homogenization buffer. Then, the immunoblotting was performed as described above except that Na^+^, K^+^-ATPase (NKA; α5, Developmental Studies Hybridoma Bank, Iowa City, IA, USA) was used as the loading control.

### H_2_O_2_ content assay

For quantification of H_2_O_2_, 50 mg of milkfish livers were homogenized in PBS solution and then centrifuged for 15 min at 1000 × *g*. The H_2_O_2_ contents were subsequently determined using the hydrogen peroxide colorimetric/fluorometric assay kit (Biovision, Bilpitas, CA, USA). We added 10 μL samples into each well of the 96-well plate, and brought the volume to 50 μL with the H_2_O_2_ assay buffer. Different concentrations of H_2_O_2_ (0, 1, 2, 3, 4, 5 nmol well^−1^) were used as the standard for calculation. The VERSAmax microplate reader (Molecular Devices, Sunnyvale, CA, USA) with the wavelength of OD_570nm_ was used to measure the H_2_O_2_ content.

### *Ex vivo* H_2_O_2_ induced *Ccprdx6* expression

Milkfish liver tissues were prepared according to Tang et al. (2012) with minor modification. The livers from SW-acclimated milkfish were excised and incubated in sterile 35-mm petri dishes containing Leibovitz-15 (L-15) medium. The culture medium Leibovitz-15 (L-15) containing 300 μg mL^−1^ L-glutamine and 4 mg mL^−1^ BSA with 500 U mL^−1^ penicillin and 500 μg mL^−1^ streptomycin were added to the petri dish before use. Milkfish liver tissues were pre-incubated in the culture medium at 28°C for 2 h. Then, the liver tissues were transferred to L-15 medium with or without 20 μM or 40 μM H_2_O_2_. After 24 h, incubation was terminated by freezing the liver tissues in liquid nitrogen. Total RNA extraction and quantitative real-time PCR were performed as described above.

### Statistical analysis

The quantitative values from 1-week data (168 h) from hypothermal and control groups were compared using a two-way analysis of variance (ANOVA) followed by Student's *t*-test post hoc method. The values were expressed as the mean ± standard error of the mean (S.E.M.), and a *p* < 0.05 was set as the significance level. Data from the time-course experiments and the *ex vivo* H_2_O_2_ induced *Ccprdx6* expression experiments were compared using one-way ANOVA analyses with Dunnett's pairwise method and Tukey's pairwise method, respectively, and a *p* < 0.05 was set as the significance level.

## Results

### Identification and characterization of *Ccprdx6* cDNA sequence

**Figure 1:**
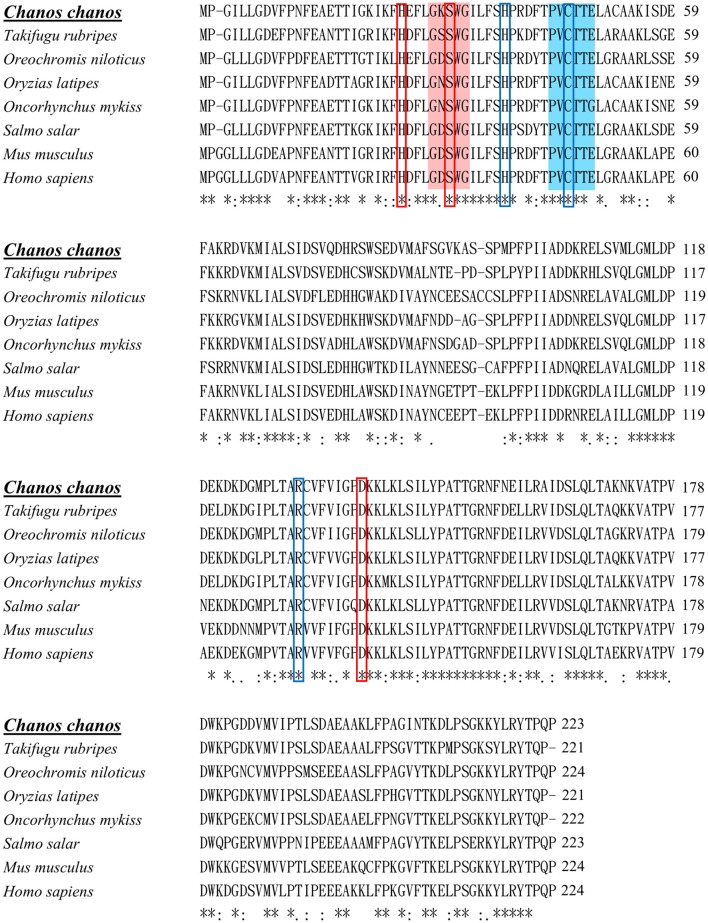
**Multiple sequence alignment of Prdx6s of different vertebrates**. Identical residues in all sequences are indicated by asterisks (^*^), conserved substitutions are indicated by colons (:), and semi-conserved substitutions are indicated by dots (.) under the column. Deletions are indicated by dashes. The peroxidase catalytic center is highlighted with blue color. Conserved amino acid residues in peroxidase catalytic triad are indicated by blue colored boxes. The conserved active site for phospholipase A_2_ is highlighted with red color. Conserved amino acid residues in phospholipase A_2_ triad are indicated by red colored boxes. Sequence alignment was performed by clustalW (1.2.1). The accession numbers of sequences were listed in Table 1.

**Figure 2:**
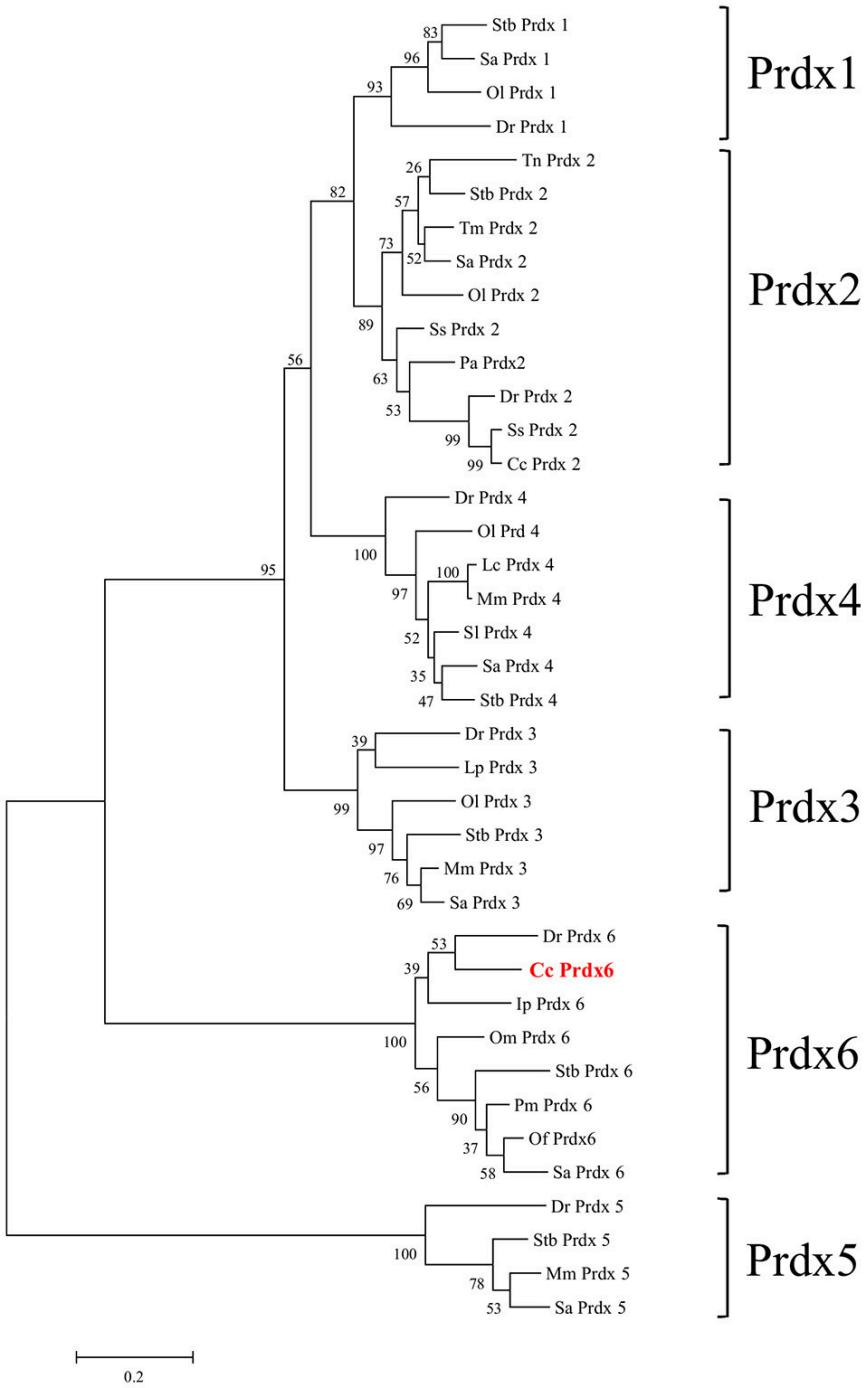
**Phylogenetic analysis of peroxiredoxin (Prdx) amino acid family sequences**. The putative Prdx protein sequences of other species were obtained from the NCBI database. The results were confirmed by 1000 bootstraps.

Figure 1 shows the 974-bp full-length *Ccprdx6* cDNA encoding 223 amino acids identified from livers of milkfish by Rapid amplification of cDNA ends (RACE)-PCR. The cDNA sequence contained a 5′-untranslated region (UTR) of 15 bp, an open reading frame (ORF) of 672 bp, and a 3′-UTR of 287 bp. The ORF encoded 223 amino acids with a molecular weight of 24.6 kDa and theoretical isoelectric point (pI) of 5.96. The predicted CcPrdx6 protein exhibited the typical 1-cysteine peroxiredoxin domains, including the peroxiredoxin center (^44^PVCTTE^49^) with a catalytic active site cysteine residue (C^46^, Figure 1). The catalytic triad residues involved in peroxidase (H^38^, C^46^, and R^131^) and phospholipase A_2_ (H^25^, S^31^, and D^139^) activity were also identified. The highly conserved amino acid sequences were observed in the entire Prdx6 sequence. The results of pair-wise amino acid comparison showed the deduced CcPrdx6 amino acid sequence was followed by *Oncorhynchus mykiss* (84.8%), *Oryzias latipes* (84.3%), *Takifugu rubripes* (81.2%), *Oreochromis niloticus* (74.1%), *Salmo salar* (74.0%), *Homo sapiens* (71.9%), and *Mus musculus* (70.5%) (Table 1). According to the phylogenetic tree, different prdx members selected from seven species of teleosts clearly indicated six subgroups of prdx, as has been published for mammals (Figure 2). The milkfish Prdx6 sequence was categorized in the Prdx6 subgroup and was closely related to zebrafish (*Danio rerio*) Prdx6.

**Table 1:** **Pairwise identity and similarity percentages of CcPrdx6 with selected Prdx6 amino acid sequences**.

**Species**	**Accession number**	**Identity (%)**	**Similarity (%)**	**Amino acids**
*Takifugu rubripes*	XP_012695082	81.2	89.7	221
*Orechromis niloticus*	XP_005458127	74.1	87.1	224
*Oryzias latipes*	XP_004068220	84.3	91.0	221
*Oncorhynchus mykiss*	NP_001158604	84.8	91.5	222
*Salmo salar*	XP_013996229	74.0	88.8	223
*Mus musculus*	AAP21829	70.5	84.4	224
*Homo sapiens*	NP_004896	71.9	86.2	224

### Tissue expression of *Ccprdx6*

**Figure 3:**
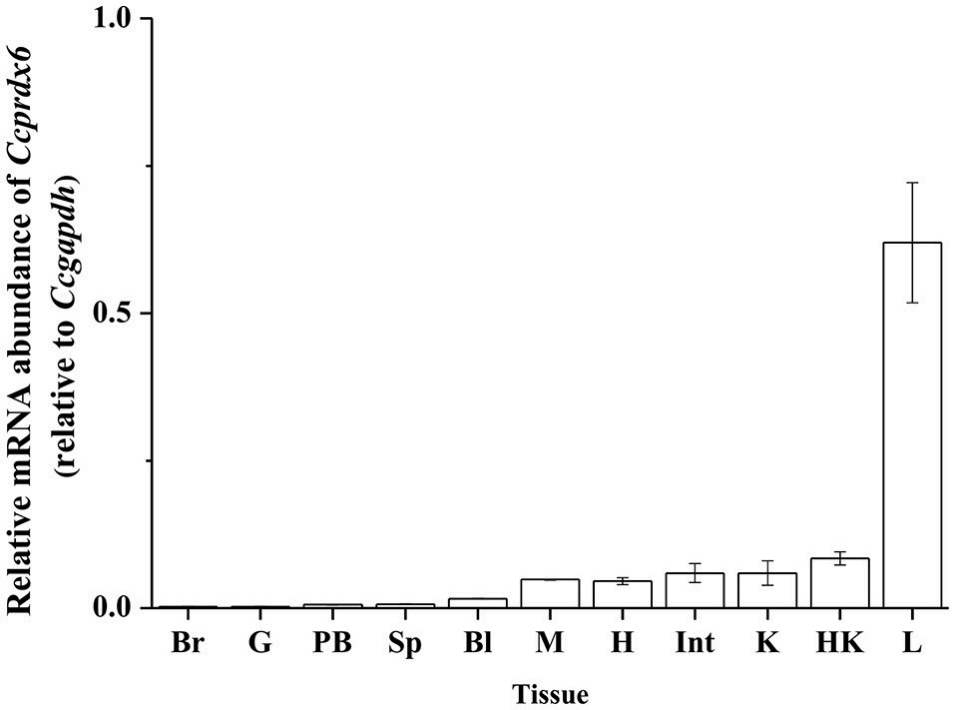
**Tissue distribution of ***Ccprdx6*** mRNA expression detected by qPCR in milkfish**. Values are means ± SEM (*n* = 3 for all groups).

To determine the tissue expression profile of *Ccprdx6* in seawater (SW)-acclimated milkfish, total RNA was extracted from the brain, gill, pseudobranch, spleen, blood, muscle, heart, intestine, kidney, head kidney, and liver for qPCR analysis. The *Ccprdx6* expression level was normalized to *Ccgapdh*. The same internal control was used to determine the tissue expression profile (Figure 3). The highest *Ccprdx6* expression level was found in the liver.

### Low-temperature effects on liver *Ccprdx6* expression

**Figure 4:**
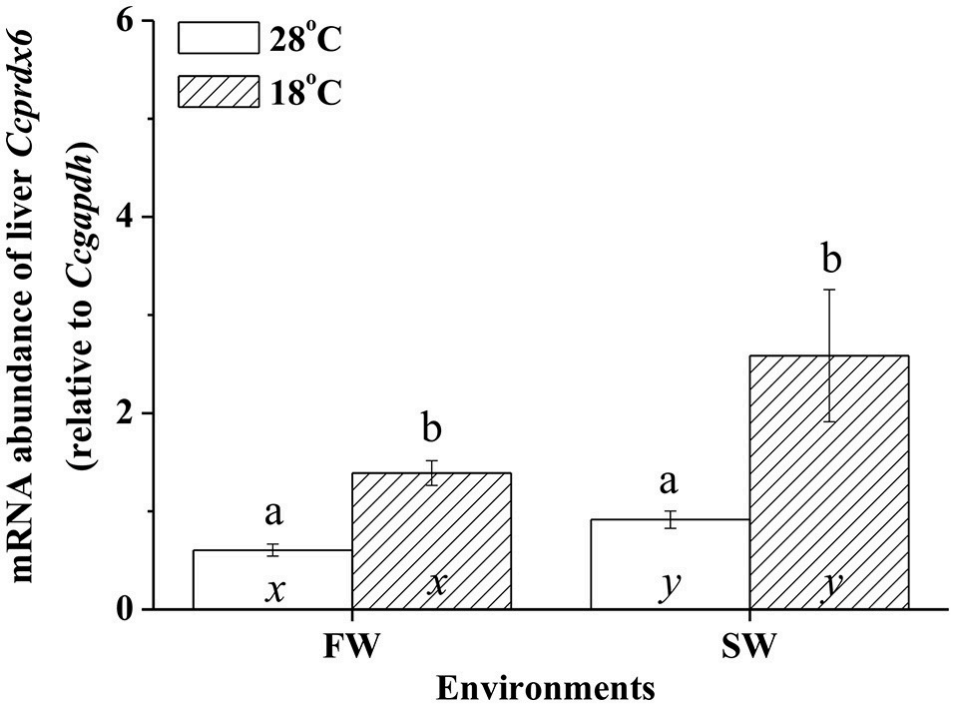
**The expression of hepatic ***Ccprdx6*** of the fresh water (FW) and seawater (SW) milkfish acclimated to 28°C (white bar) or 18°C (stripe bar) for 1 week, respectively**. Values are means ± SEM, *n* = 6. Different letters (a and b) indicate significant differences between the 28° and 18°C group, and (x and y) indicate significant differences between the FW and SW group. The Student's *t*-test pairwise comparison was used following two-way ANOVA, *P* < 0.05.

**Figure 5:**
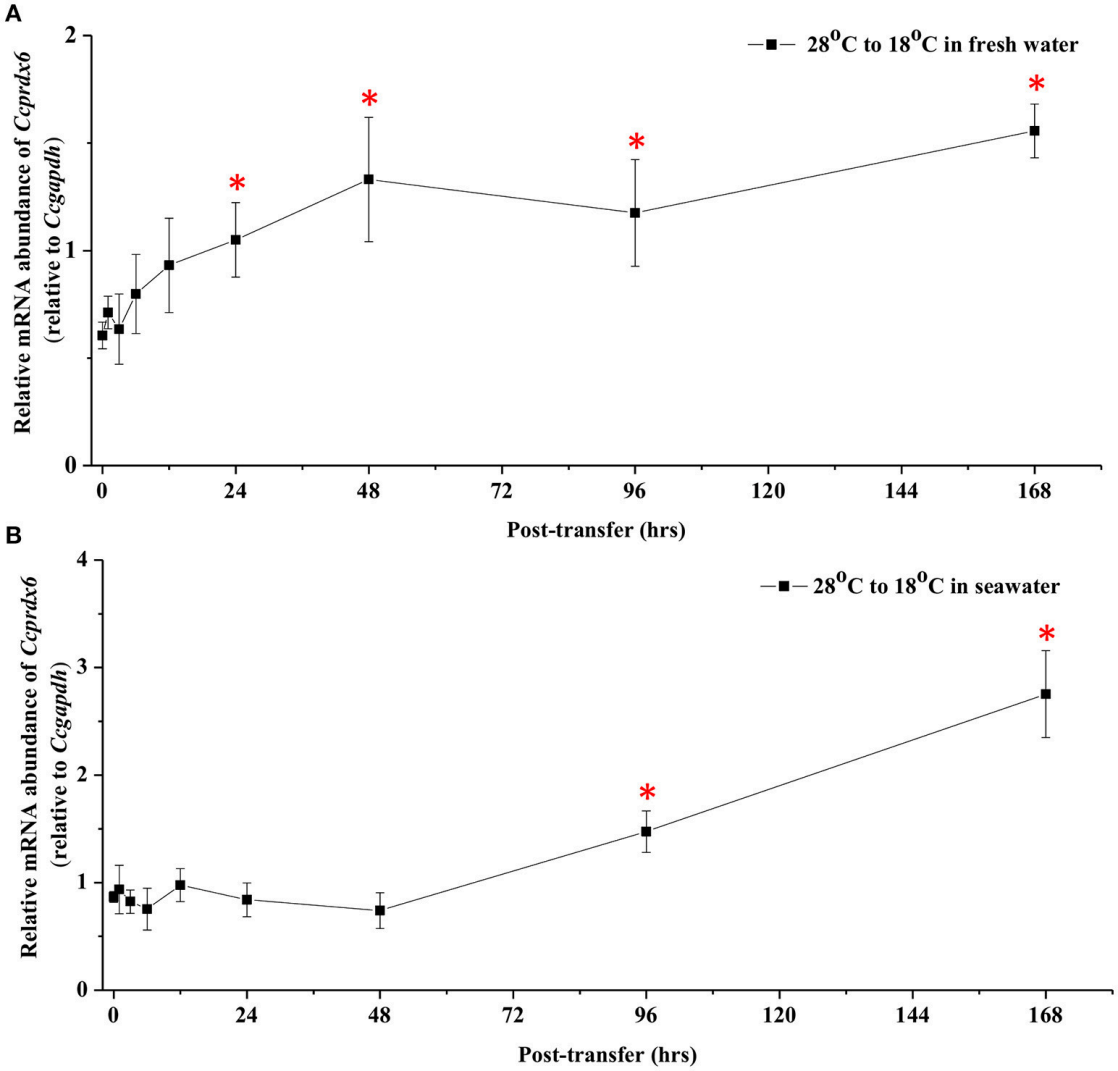
**Time-course expression of ***Ccprdx6*** mRNA in livers of (A)** FW and **(B)** SW milkfish after transferred from 28° to 18°C. Values were normalized by GAPDH. Values are means ± SEM, *n* = 6. The asterisk indicates significant differences compared to the 0 h fish by one-way ANOVA (Dunnet's comparison, *P* < 0.05).

In fresh water (FW)- and SW-acclimated milkfish, the liver *Ccprdx6* expression was up-regulated under hypothermal stress (Figure 4). The two-way ANOVA analyses revealed that *Ccprdx6* expression was affected by hypothermal stress [*F*_(1, 23)_ = 12.54, *p* = 0.002] and salinity challenge [*F*_(1, 23)_ = 4.73, *p* = 0.042]. The synergistic interaction between temperature and salinity did not significantly affect *Ccprdx6* expression [*F*_(1, 23)_ = 1.63, *p* = 0.216]. The time-course *Ccprdx6* expression in livers was determined by qPCR at 1, 3, 6, 12, 24, 48, 96, and 168 h after transfer to 18°C in FW (Figure 5A) or SW (Figure 5B). Compared to the 0 h controls (fish at 28°C), the mRNA abundance of *Ccprdx6* increased significantly after 24 h in FW (Figure 5A) and 96 h in SW (Figure 5B), post-hypothermal challenge.

### The protein expression of Ccprdx6 upon hypothermal challenge

**Figure 6:**
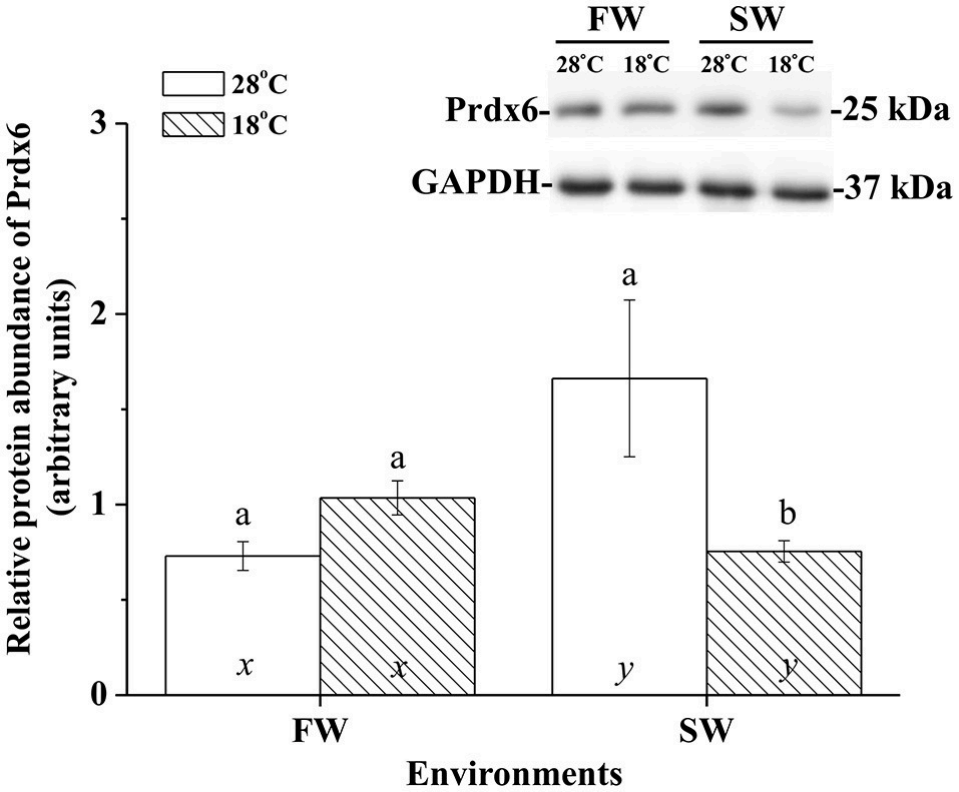
**Relative abundance of Prdx6 proteins in livers of the fresh water (FW) and seawater (SW) milkfish acclimated to 28°C (white bar) and 18°C (stripe bar) for 1 week, respectively**. The representative immunoblots showed single immunoreactive band of Prdx6 at about 25 kDa. The single immunoreactive band at 37 kDa was GAPDH used as the loading control. Different letters (a and b) indicate significant differences between the 28° and 18°C group, and (x and y) indicate significant differences between the FW and SW group. Values are means ± SEM, *n* = 6. The Student's *t*-test pairwise comparison was used following two-way ANOVA, *P* < 0.05.

**Figure 7:**
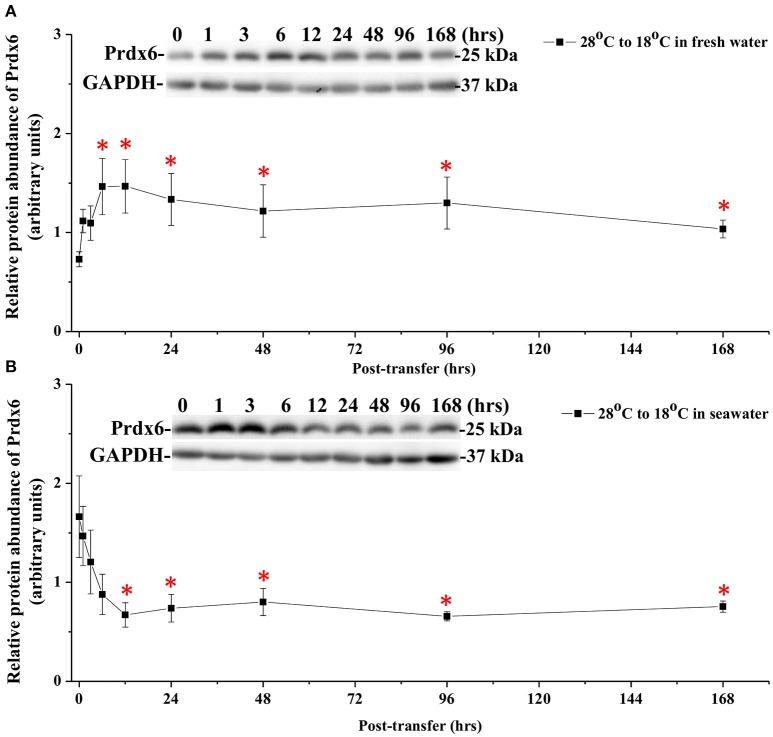
**Time-course expression of Prdx6 protein in livers of (A)** FW and **(B)** SW milkfish after transferred from 28° to 18°C. The representative immunoblots indicate the immunoreactive bands of Prdx6 probed with a polyclonal antibody. GAPDH was used as the loading control. Values are means ± SEM, *n* = 6. The asterisks indicate significant differences by one-way ANOVA (Dunnet's comparison, *P* < 0.05).

The effects of hypothermal stress on CcPrdx6 protein abundance were determined by immunoblots. CcPrdx6 was detected as a single immunoreactive band at 25 kDa. At normal temperature (28°C), higher protein abundance of CcPrdx6 was found in the SW group than the FW group (Figure 6). In the low-temperature (18°C) group compared to the normal-temperature group, however, protein abundance of hepatic CcPrdx6 was not significantly changed in the FW-acclimated milkfish but significantly down-regulated in the SW-acclimated milkfish (Figure 6). The two-way ANOVA analyses revealed that neither hypothermal stress [*F*_(1, 23)_ = 4.40, *p* = 0.189] nor salinity challenge [*F*_(1, 23)_ = 4.88, *p* = 0.157] affected CcPrdx6 protein expression, whereas the synergistic interaction between temperature and salinity [*F*_(1, 23)_ = 18.55, *p* = 0.016] significantly affected CcPrdx6 protein expression. After transfer from 28° to 18°C, hepatic CcPrdx6 protein expression was significantly up-regulated from 6 h to 7 days in the FW group (Figure 7A). In the SW group, however, hepatic CcPrdx6 protein abundance was down-regulated from 12 h to 7 days (Figure 7B).

### Expression of membrane Ccprdx6 protein under hypothermal stress

**Figure 8:**
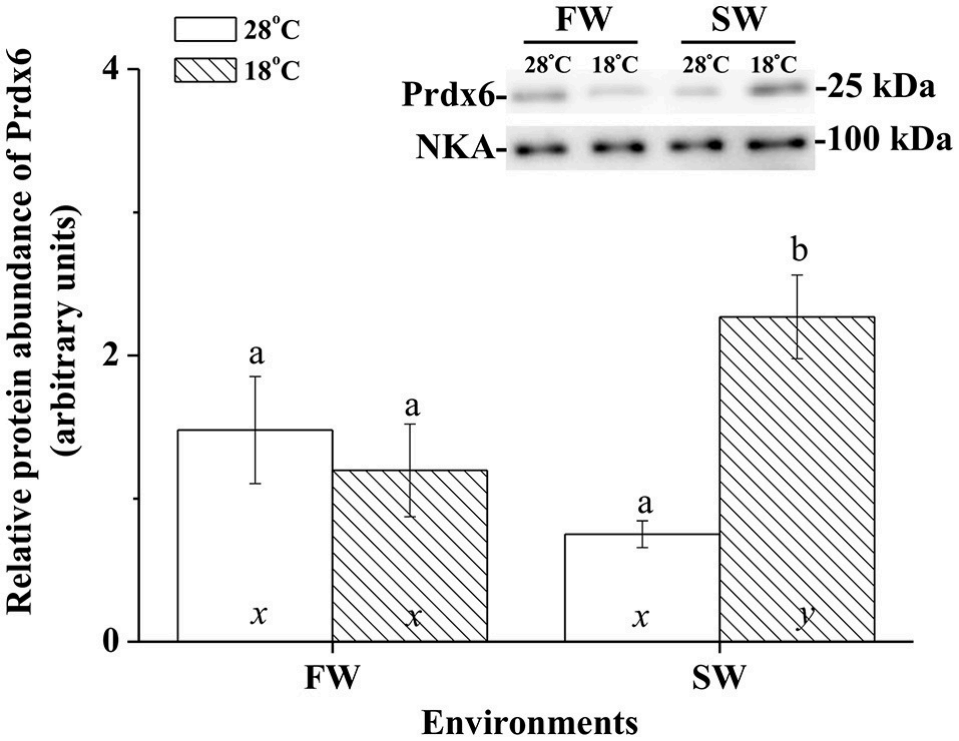
**The immunoblots and relative intensities of Prdx6 in membrane-fraction proteins of livers of the fresh water (FW) and seawater (SW) milkfish acclimated to 28°C (white bar) and 18°C (stripe bar) for 1 week, respectively**. In the representative immunoblot, single immunoreactive bands at 100 and 25 kDa represent Na^+^, K^+^-ATPase (NKA), and Prdx6, respectively. The NKA was used as the loading control of the membrane fraction. Different letters (a and b) indicate significant differences between the 28° and 18°C group, and (x and y) indicate significant differences between the FW and SW group. Values are means ± SEM, *n* = 6. The Student's *t*-test pairwise comparison was used following two-way ANOVA, *P* < 0.05.

Immunoblotting of membrane fraction of milkfish livers using the Na^+^, K^+^-ATPase (NKA) as the loading control showed a single immunoreactive band of CcPrdx6 at 25 kDa. In FW-acclimated milkfish, relative amounts of the CcPrdx6 in membrane fraction proteins were not significantly different between the normal- and low-temperature groups. In SW-acclimated milkfish livers, however, membrane CcPrdx6 was up-regulated in the low-temperature group, but not in the control group. Moreover, expression of membrane CcPrdx6 protein was significantly higher in the hypothermal SW group than in the hypothermal FW group (Figure 8). The two-way ANOVA analyses revealed that hypothermal stress [*F*_(1, 23)_ = 5.40, *p* = 0.031], but not salinity challenge [*F*_(1, 23)_ = 0.48, *p* = 0.497], affected expression of membrane CcPrdx6. The synergistic interaction between temperature and salinity also significantly affected expression of membrane CcPrdx6 [*F*_(1, 23)_ = 11.14, *p* = 0.003].

### H_2_O_2_ contents in milkfish livers under hypothermal stress

**Figure 9:**
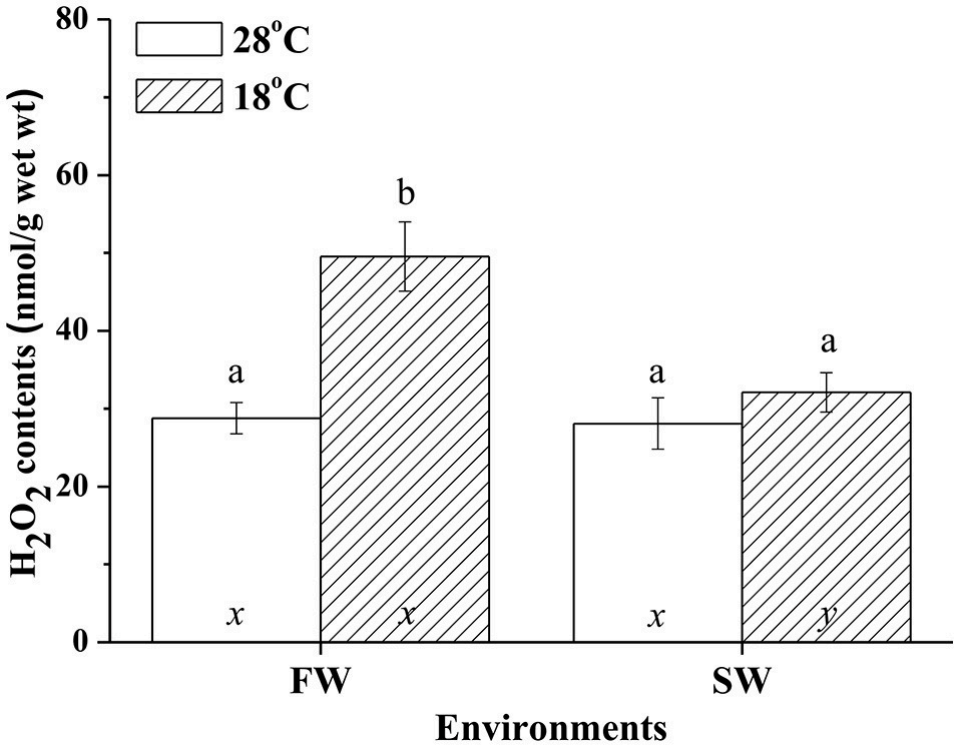
**H_**2**_O_**2**_ contents in livers of fresh water (FW) and seawater (SW) milkfish acclimated to 28°C (white bar) and 18°C (stripe bar) for 1 week, respectively**. Different letters (a and b) indicate significant differences between the 28° and 18°C group, and (x and y) indicate significant differences between the FW and SW group. Values are means ± SEM, *n* = 6. The Student's *t*-test pairwise comparison was used following two-way ANOVA, *P* < 0.05.

Hypothermal stress induced H_2_O_2_ in livers of FW but not SW milkfish. In the SW milkfish, similar H_2_O_2_ contents were found between the normal and low-temperature group. In addition, under hypothermal stress, hepatic H_2_O_2_ contents of the FW-acclimated milkfish were significantly higher than in the SW-acclimated group (Figure 9). The two-way ANOVA analyses revealed that hypothermal stress [*F*_(1, 23)_ = 14.99, *p* = 0.001], salinity challenge [*F*_(1, 23)_ = 8.04, *p* = 0.010], and the synergistic interaction between temperature and salinity [*F*_(1, 23)_ = 6.87, *p* = 0.016] significantly affected hepatic H_2_O_2_ content.

### *Ex vivo Ccprdx6* expression in response to oxidative stress

**Figure 10:**
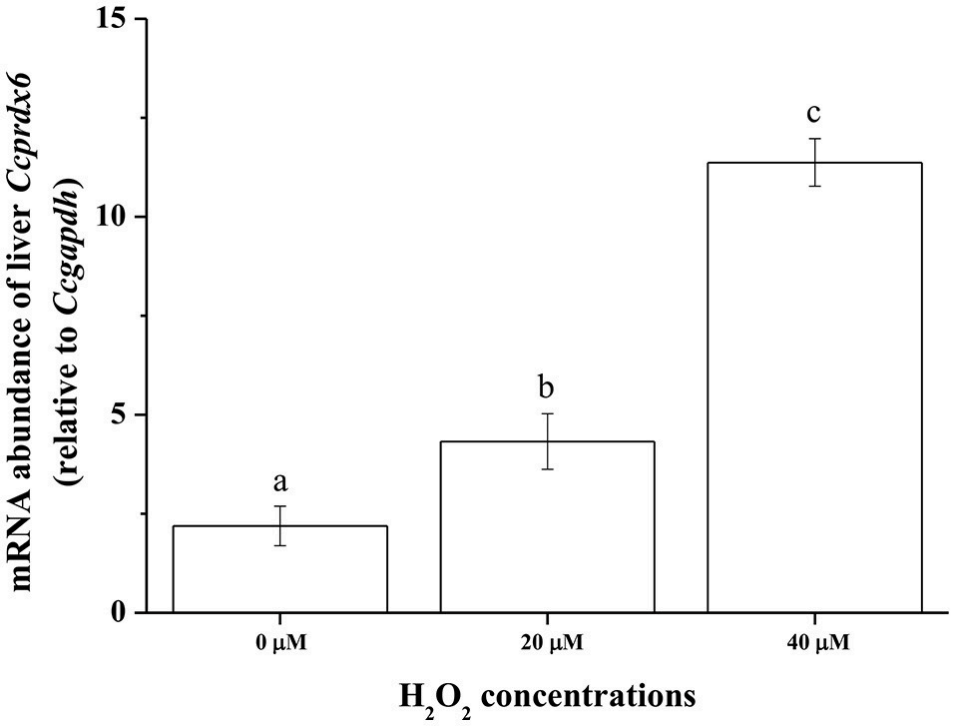
**H_**2**_O_**2**_ induced ***ex vivo Ccprdx6*** expression of milkfish liver tissues**. Tissue cultures of milkfish livers were treated with different concentrations of H_2_O_2_. Values were normalized by GAPDH. Different letters indicated significant differences compared with 0 μM using one-way ANOVA (Tukey's pairwise comparison, *P* < 0.05). Values were means ± SEM, *n* = 4.

To examine *Ccprdx6* expression under oxidative stress induced by H_2_O_2_, *ex vivo* tissue cultures of milkfish livers were exposed to different concentrations of H_2_O_2_ for 24 h. qPCR analysis showed that *Ccprdx6* expression in cultured liver tissues increased with H_2_O_2_ concentrations in the medium (Figure 10).

## Discussion

Multiple sequence alignment revealed that the deduced amino acid sequence of CcPrdx6 exhibited more than 60% identity compared with the Prdx6 of other teleosts and mammals (81.2% with *T. rubripes*, 84.8% with *O. mykiss*, 84.3% with *O. latipes*, 74.1% with *O. niloticus*, 74.0% with *S. salar*, 71.9% with *H. sapiens*, and 70.5% with *M. musculus*). CcPrdx6 contained sequences of conserved regions and catalytic triads involved in peroxidase and PLA_2_ domain regions (Choi et al., 1998; Fisher, 2011). The peroxidase activity catalytic triad (Cys_46_-His_38_-Arg_131_) and PLA_2_ activity catalytic triad (Ser_31_-His_25_-Asp_139_) of CcPrdx6 are highly conserved compared with those of other mammals and teleosts. In the sequences of 1-Cysteine cluster in peroxidase catalytic center (^44^PVCTTE^49^) and the lipase motif (^29^GXSXG^33^), Prdx6 was classified as a member of 1-Cys peroxiredoxin in the Prdx protein family (Zheng et al., 2010; Fisher, 2011; De Zoysa et al., 2012; Mu et al., 2013; Priyathilaka et al., 2016; Tolomeo et al., 2016). Previous studies revealed that in human Prdx6 sequence, Cys_47_ was bound to His_39_ by the hydrogen bond and electrostatically activated by Arg_132_. In addition, the Ser_32_ in the motif for PLA_2_ activity of the human Prdx6 sequence is crucial for modulation of activity (Manevich and Fisher, 2005; Fisher, 2011). An *ex vivo* study further demonstrated that when the pH of culture medium decreased to 4, phosphorylation of human Prdx6 increased, and PLA_2_ activity of human Prdx6 increased to more than 10 fold (Wu et al., 2009). The conserved phosphorylation site at Thr_177_ of the Prdx6 sequence first identified by the mass spectroscopic analysis in the rat was also found in the milkfish and other species. Highly conserved active residues of CcPrdx6 compared with the sequences of mammalian Prdx6 indicated that they might have the same biological functions, as well as phosphorylation site to regulate enzyme activity.

In mammals, Prdx6 is expressed in all major tissues, including the liver, lung, heart, and spleen. The expression profiles in these organs suggested the role of Prdx6 in maintaining ROS balance (Da Silva-Azevedo et al., 2009; Fisher, 2011). In the gilthead seabream (*Sparus aurata*), gene expression of Prdx1–6 was identified in 11 tissues. Prdx members were highly expressed in livers of the gilthead seabream because the liver might be exposed to oxidative stress by the products of metabolic reaction (Pérez-Sánchez et al., 2011). The *prdx6* mRNA was ubiquitously expressed in all detected tissues of the other teleosts, such as the Japanese eel (*Anguilla japonica*; Priyathilaka et al., 2016), turbot (*Scophthalmus maximus*; Zheng et al., 2010), and Antarctic emerald rockcod (*Trematomus Bernacchii*; Tolomeo et al., 2016). Furthermore, high abundance of *prdx6* mRNA was found in livers of rock bream (*Acanthopagrus butcheri*) and *prdx6* mRNA expression changed with poly I:C treatment or iridovirus challenge (De Zoysa et al., 2012). On the other hand, studies on the Japanese eel and turbot focused on antioxidant functions of hepatic Prdx6 against poly I:C and bacteria challenge (Zheng et al., 2010; Priyathilaka et al., 2016). Studies on the Antarctic emerald rockcod also focused on hepatic *prdx6* expression in response to warming environments (Tolomeo et al., 2016). In this study, the milkfish *prdx6* was expressed in 11 organs and was most abundant in the liver. Taken together, highly expressed Prdx6 in milkfish livers suggested its potential roles as an antioxidant in metabolizing ROS and protection from increasing oxidative stress induced by hypothermal environments.

The oxidative stress of fish arose when fish were placed in environments outside the optimal range of temperature, whether it was heat stress or hypothermal challenge (Pörtner et al., 2007). Oxidative stress, as well as thermal stress threatened to damage the structures of molecules. The anti-oxidant mechanism, anaerobic metabolism, and molecular chaperones, however, were regulated upon temperature fluctuation (Pörtner et al., 2007). This study revealed that H_2_O_2_ content was elevated in livers of FW-acclimated milkfish indicating an increase in oxidative stress upon hypothermal challenge. Kammer et al. (2011) found that oxidative stress of the three spine stickleback (*Gasterosteus aculeatus*), determined by measuring the protein carbonyls, glutathione index, and transcript levels of superoxide dismutase, increased when fishes were acclimated to warm (20°C) or cold (8°C) environments. Total ROS and lipid peroxidation were also increased upon cold shock in the liver, brain, and gill of the zebrafish (Wu et al., 2015). In addition, lipid peroxidation and NO production increased in livers of the gilthead seabream under cold conditions (Ibarz et al., 2010). Two-way ANOVA analyses in the present study further illustrated that both environmental temperatures and salinities were important factors for changes in the oxidative stress (indicated by H_2_O_2_ content) of milkfish livers. Moreover, *ex vivo* H_2_O_2_ treatment induced dose-dependent *Ccprdx6* expression in cultured liver tissues of milkfish. Hence, it is reasonable to infer that *Ccprdx6* expression increased in response to low-temperature induced oxidative stress in livers of hypothermal FW- and SW-acclimated milkfish.

Analysis of the gilthead sea bream liver transcriptome revealed immediate and sustained activation of NFE2L2/NRF2, the main antioxidant response transcription factor, under cold stress (Mininni et al., 2014). The gene expression of antioxidant enzymes (SOD, GPx, and Cat) was also up-regulated upon acute hypothermal challenge in zebrafish livers (Wu et al., 2015). In the present study, significant up-regulation in *prdx6* transcript levels was found in both 18°C FW and SW groups. In addition, *ex vivo* H_2_O_2_ treatments on milkfish livers also led to up-regulation of *Ccprdx6* after 24 h incubation, implying that prdx6 gene expression increased in response to oxidative stress upon hypothermal challenge. On the other hand, changes in environmental salinities were found to be associated with the enhancement of ROS generation, whereas imbalance of the antioxidant mechanisms induced oxidative damage in aquatic organisms (Martínez-Alvarez et al., 2002; Paital and Chainy, 2010; Lushchak, 2011). Acute up-regulation of both enzymatic and non-enzymatic antioxidant mechanisms were reported in fish to neutralize ROS damage against the oxidative stress (Mininni et al., 2014; Nakano et al., 2014; Wu et al., 2015). Therefore, salinity and hypothermal stress together might enhance ROS generation and induce oxidative damage in FW low-temperature milkfish, leading to more acute up-regulation of *prdx6* in the FW group (24 h) than the SW group (96 h) upon hypothermal challenge. Upon hypothermal challenge, FW-acclimated milkfish might encounter more ROS (indicated by H_2_O_2_ content), which induced *Ccprdx6* expression after 24 h of low-temperature exposure.

Two functional domains of mammalian Prdx6 protein were identified, i.e., the peroxidase and PLA_2_ catalytic regions (Manevich and Fisher, 2005; Fisher, 2011). This bi-functional protein plays the role of peroxidase in the cytosol and reduces oxidization of phospholipids in the membrane (Da Silva-Azevedo et al., 2009; Fisher, 2011). The mass spectrometry analysis of the rat revealed that the phosphorylation site was at Thr177, which enables a 10-fold increase of PLA_2_ activity (Wu et al., 2009). Under oxidative stress, the 2D-PAGE analysis identified that the pI of prdx6 was shifted from 6.5 to 5.9 in the skeletal muscle of knockout mice lacking nNOS (Da Silva-Azevedo et al., 2009). In addition, the influence of heat stress on membrane and cytosolic proteins in human erythrocytes was analyzed by electrophoresis and Prdx6 protein was found to be up-regulated in the membrane fraction in subsequent mass spectrometry analyses (Sharma et al., 2013). Our previous proteomic study found that pI of milkfish Prdx6 protein spots was shifted from 6.35 to 6.19 in livers of the non-lethal and lethal group, respectively (Chang et al., 2016). This study further revealed that Prdx6 protein expression was up-regulated after 6 h hypothermal challenge in FW-acclimated milkfish. Because H_2_O_2_ can be neutralized by Prdx6 in the cytosol (Fisher, 2011), milkfish Prdx6 protein may play the role of antioxidant enzyme through neutralizing oxidative stress in the cytosol of hepatocytes. On the other hand, the oxidative stress led to phospholipid peroxidation, and the major substrates are polyunsaturated fatty acids containing more than one double bond in their backbone. PUFAs play major roles in affecting cell membrane fluidity, which is particularly important in physiological responses upon hypothermal challenge. A continued peroxidation state of lipids led to the loss of membrane fluidity, and hence decreased the functions of membrane proteins (Pörtner et al., 2007; Fisher, 2011; de la Haba et al., 2013). Accordingly, in livers of SW-acclimated milkfish, abundance of membrane CcPrdx6 protein was significantly higher in the hypothermal group than in the control group indicating its potential role as an antioxidant through binding to membranes to stabilize the membrane redox state.

In conclusion, this study identified the expression and molecular characterization of the antioxidant, Ccprdx6, at transcriptional and protein levels in milkfish livers. *Ex vivo* H_2_O_2_ treatments of milkfish liver tissues induced a dose-dependent increase in *Ccprdx6* mRNA abundance. Under hypothermal environments, different redox states between livers of SW- and FW-acclimated milkfish indicated different oxidative stress between the SW and FW groups. More oxidative stress occurred in hypothermal FW milkfish, rather than hypothermal SW milkfish, and this may be caused by synergistic effects from both salinity and temperature changes. In response to different oxidative stress between SW and FW milkfish livers upon hypothermal challenge, CcPrdx6 was found to express through different mechanisms to play the role of an antioxidant. In the FW group, Ccprdx6 protein was up-regulated for H_2_O_2_ scavenging in the cytosol. In the SW group, however, the CcPrdx6 protein was translocated to the membrane for maintaining the redox state and stabilizing lipid structure.

## Author contributions

CC and TL conceived and designed the research. CC carried out the experiments. CC wrote the original manuscript. WL and TL reviewed and edited the manuscript. TL supervised the project. All authors have approved the manuscript for publication.

### Conflict of interest statement

The authors declare that the research was conducted in the absence of any commercial or financial relationships that could be construed as a potential conflict of interest. The reviewer MZ and handling Editor declared their shared affiliation, and the handling Editor states that the process nevertheless met the standards of a fair and objective review.

## References

[B1] Abele D. Puntarulo S. (2004). Formation of reactive species and induction of antioxidant defence systems in polar and temperate marine invertebrates and fish. Comp. Biochem. Physiol. A Mol. Integr. Physiol. 138, 405–415. 10.1016/j.cbpb.2004.05.013 15369829

[B2] Bagarinao T. (1994). Systematics, distribution, genetics and life history of milkfish, *Chanos chanos*. Environ. Biol. Fishes 39, 23–41. 10.1007/BF00004752

[B3] Chang C. H. Tang C. H. Kang C. K. Lo W. Y. Lee T. H. (2016). Comparison of integrated response to nonlethal and lethal hypothermal stress in milkfish (*Chanos chanos*): a proteomics study. PLoS ONE 11:e0163538. 10.1371/journal.pone.0163538 27657931 PMC5033585

[B4] Choi H. I. Ma S. K. Bae E. H. Lee J. Kim S. W. (2016). Peroxiredoxin 5 protects TGF-β induced fibrosis by inhibiting Stat3 activation in rat kidney interstitial fibroblast cells. PLoS ONE 11:e0149266. 10.1371/journal.pone.0149266 26872211 PMC4752225

[B5] Choi H. J. Kang S. W. Yang C. H. Rhee S. G. Ryu S. E. (1998). Crystal structure of a novel human peroxidase enzyme at 2.0 Å resolution. Nat. Struct. Biol. 5, 400–406. 10.1038/nsb0598-400 9587003

[B6] Choi K. J. Kim M. J. Je A. R. Jun S. Lee C. Lee E.. (2014). Three-dimensional analysis of abnormal ultrastructural alteration in mitochondria of hippocampus of APP/PSEN1 transgenic mouse. J. Biosci. 39, 97–105. 10.1007/s12038-013-9406-8 24499794

[B7] Da Silva-Azevedo L. Jähne S. Hoffmann C. Stalder D. Heller M. Pries A. R.. (2009). Up-regulation of the peroxiredoxin-6 related metabolism of reactive oxygen species in skeletal muscle of mice lacking neuronal nitric oxide synthase. J. Physiol. 587, 655–668. 10.1113/jphysiol.2008.164947 19047200 PMC2670087

[B8] de la Haba C. Palacio J. R. Martínez P. Morros A. (2013). Effect of oxidative stress on plasma membrane fluidity of THP-1 induced macrophages. Biochim. Biophys. Acta 1828, 357–364. 10.1016/j.bbamem.2012.08.013 22940500

[B9] De Zoysa M. Ryu J. H. Chung H. C. Kim C. H. Nikapitiya C. Oh C.. (2012). Molecular characterization, immune responses and DNA protection activity of rock bream (*Oplegnathus fasciatus*), peroxiredoxin 6 (Prx6). Fish Shellfish Immunol. 33, 28–35. 10.1016/j.fsi.2012.03.029 22484606

[B10] Dong W. R. Xiang L. X. Shao J. Z. (2007). Cloning and characterisation of two natural killer enhancing factor genes (NKEF-A and NKEF-B) in pufferfish, *Tetraodon nigroviridis*. Fish Shellfish Immunol. 22, 1–15. 10.1016/j.fsi.2006.03.007 16690325

[B11] Fisher A. B. (2011). Peroxiredoxin 6: a bifunctional enzyme with glutathione peroxidase and phospholipase A_2_Ć activities. Antioxid. Redox Signal. 15, 831–844. 10.1089/ars.2010.3412 20919932 PMC3125547

[B12] Heise K. Puntarulo S. Nikinmaa M. Abele D. Pörtner H. O. (2006). Oxidative stress during stressful heat exposure and recovery in the North Sea eelpout *Zoarces viviparous*. J. Exp. Biol. 209, 353–363. 10.1242/jeb.01977 16391357

[B13] Hermes-Lima M. Zenteno-Savín T. (2002). Animal response to drastic changes in oxygen availability and physiological oxidative stress. Comp. Biochem. Physiol. C 133, 537–556. 10.1016/s1532-0456(02)00080-7 12458182

[B14] Hsieh S. L. Kuo C. M. (2005). Stearoyl–CoA desaturase expression and fatty acid composition in milkfish (*Chanos chanos*) and grass carp (*Ctenopharyngodon idella*) during cold acclimation. Comp. Biochem. Physiol. B 141, 95–101. 10.1016/j.cbpc.2005.02.001 15820139

[B15] Hu Y. C. Kang C. K. Tang C. H. Lee T. H. (2015). Transcriptomic analysis of metabolic pathways in milkfish that respond to salinity and temperature changes. PLoS ONE 10:e0134959. 10.1371/journal.pone.0134959 26263550 PMC4532362

[B16] Ibarz A. Martín-Pérez M. Blasco J. Bellido D. de Oliveira E. Fernández-Borràs J. (2010). Gilthead sea bream liver proteome altered at low temperatures by oxidative stress. Proteomics 10, 963–975. 10.1002/pmic.200900528 20131326

[B17] Kammer A. R. Orczewska J. I. O'Brien K. M. (2011). Oxidative stress is transient and tissue specific during cold acclimation of threespine stickleback. J. Exp. Biol. 214, 1248–1256. 10.1242/jeb.053207 21430200

[B18] Kang C. K. Chen Y. C. Chang C. H. Tsai S. C. Lee T. H. (2015). Seawater-acclimation abates cold effects on Na+, K+-ATPase activity in gills of the juvenile milkfish, *Chanos chanos*. Aquaculture 446, 67–73. 10.1016/j.aquaculture.2015.04.022

[B19] Konno T. Pinho Melo E. Lopes C. Mehmeti I. Lenzen S. Ron D.. (2015). ERO1-independent production of H_2_O_2_ within the endoplasmic reticulum fuels Prdx4-mediated oxidative protein folding. J. Cell Biol. 211, 253–259. 10.1083/jcb.201506123 26504166 PMC4621842

[B20] Lushchak V. I. (2011). Environmentally induce oxidative stress in aquatic animals. Aquat. Toxicol. 101, 13–30. 10.1016/j.aquatox.2010.10.006 21074869

[B21] Manevich Y. Fisher A. B. (2005). Peroxiredoxin 6, a 1-Cys peroxiredoxin, functions in antioxidant defense and lung phospholipid metabolism. Free Radic. Bio. Med. 38, 1422–1432. 10.1016/j.freeradbiomed.2005.02.011 15890616

[B22] Manevich Y. Hutchens S. Tew K. D. Townsend D. M. (2013). Allelic variants of glutathione S-transferase P1-1 differentially mediate the peroxidase function of Peroxiredoxin VI and alter membrane lipid peroxidation. Free Radic. Bio. Med. 54, 62–70. 10.1016/j.freeradbiomed.2012.10.556 23142420 PMC3539142

[B23] Manevich Y. Reddy K. S. Shuvarva T. Feinstein S. I. Fisher A. B. (2007). Structure and phospholipase function of peroxiredoxin 6: identification of the catalytic triad and its role in phospholipid substrate binding. J. Lipid Res. 48, 2306–2318. 10.1194/jlr.M700299-JLR200 17652308

[B24] Manevich Y. Shuvaeva T. Dodia C. Kazi D. Feinstein S. I. Fisher A. B. (2009). Binding of peroxiredoxin 6 to substrate determines differential phospholipid hydroperoxide peroxidase and phospholipase A2 activities. Arch. Biochem. Biophys. 485, 139–149. 10.1016/j.abb.2009.02.008 19236840 PMC2832859

[B25] Martínez-Alvarez R. M. Hidalgo M. C. Domezain A. Morales A. E. García-Gallego M. Sanz A. (2002). Physiological changes of sturgeon *Acipenser naccarii* caused by increasing environmental salinity. J. Exp. Biol. 205, 3699–3706. 12409496 10.1242/jeb.205.23.3699

[B26] Mininni A. N. Milan M. Ferraresso S. Petochi T. Di Marco P. Marino G.. (2014). Liver transcriptome analysis in gilthead sea bream upon exposure to low temperature. BMC Genomics 15:765. 10.1186/1471-2164-15-765 25194679 PMC4167152

[B27] Mu Y. Wan X. Lin K. Ao J. Chen X. (2013). Liver proteomic analysis of the large yellow croaker (*Pseudosciaena crocea*) following polyriboinosinic:polyribocytidylic acid induction. Fish Physiol. Biochem. 39, 1267–1276. 10.1007/s10695-013-9781-y 23479204

[B28] Nakano T. Kameda M. Shoji Y. Hayashi S. Yamaguchi T. Sato M. (2014). Effect of severe environmental thermal stress on redox state in salmon. Redox Biol. 2, 772–776. 10.1016/j.redox.2014.05.007 25009778 PMC4085342

[B29] Paital B. Chainy G. B. (2010). Antioxidant defenses and oxidative stress parameters in tissues of mud crab (*Scylla serrata*) with reference to changing salinity. Comp. Biochem. Phsiol. C 151, 142–151. 10.1016/j.cbpc.2009.09.007 19796708

[B30] Park H. Ahn I. Y. Kim H. Cheon J. Kim M. (2008). Analysis of ESTs and expression of two peroxiredoxins in the thermally stressed Antarctic bivalve *Laternula elliptica*. Fish Shellfish Immunol. 25, 550–559. 10.1016/j.fsi.2008.07.017 18723093

[B31] Pérez-Sánchez J. Bermejo-Nogales A. Calduch-Giner J. A. Kaushik S. Sitjà-Bobadilla A. (2011). Molecular characterization and expression analysis of six peroxiredoxin paralogous genes in gilthead sea bream (*Sparus aurata*): insights from fish exposed to dietary, pathogen and confinement stressors. Fish Shellfish Immunol. 31, 294–302. 10.1016/j.fsi.2011.05.015 21640832

[B32] Pörtner H. O. Peck L. Somero G. (2007). Thermal limits and adaptation in marine Antarctic ectotherms: an integrative view. Philos. Trans. R. Soc. B 362, 2333–2258. 10.1098/rstb.2006.1947 17553776 PMC2443174

[B33] Priyathilaka T. T. Kim Y. Udayantha H. M. Lee S. Herarth H. M. Lakmal H. H.. (2016). Identification and molecular characterization of peroxiredoxin 6 from Japanese eel (*Anguilla japonica*) revealing its potent antioxidant properties and putative immune relevancy. Fish Shellfish Immunol. 51, 291–302. 10.1016/j.fsi.2015.12.012 26911410

[B34] Sharma S. Zingde S. M. Gokhale S. M. (2013). Identification of human erythrocyte cytosolic proteins associated with plasma membrane during thermal stress. J. Memb. Biol. 246, 591–607. 10.1007/s00232-013-9569-0 23774970

[B35] Tang C. H. Lai D. Y. Lee T. H. (2012). Effects of salinity acclimation on Na+/K+-ATPase response and FXYD11 expression in the gills and kidneys of the Japanese eel (*Anguilla japonica*). Comp. Biochem. Physiol. A 163, 302–310. 10.1016/j.cbpa.2012.07.017 22885345

[B36] Tolomeo A. M. Carraro A. Bakiu R. Toppo S. Place S. P. Ferro D.. (2016). Peroxiredoxin 6 from the Antarctic emerald rockcod: molecular characterization of its response to warming. J. Comp. Physiol. B 186, 59–71. 10.1007/s00360-015-0935-3 26433650

[B37] Tseng Y. C. Chen R. D. Lucassen M. Schmldt M. M. Dringen R. Abele D.. (2011). Exploring uncoupling proteins and antioxidant mechanisms under acute cold exposure in brain of fish. PLoS ONE 6:e18180. 10.1371/journal.pone.0018180 21464954 PMC3064598

[B38] Wang Y. Feinstein S. I. Fisher A. B. (2008). Peroxiredoxin 6 as an antioxidant enzyme: protection of lung alveolar epithelial type II cells from H_2_O_2_-induced oxidative stress. J. Cell Biochem. 104, 1274–1285. 10.1002/jcb.21703 18260127 PMC4922305

[B39] Wood Z. A. Schröder E. Robin Harris J. Poole L. B. (2003). Structure, mechanism and regulation of peroxiredoxins. Trends Biochem. Sci. 28, 32–40. 10.1016/S0968-0004(02)00003-8 12517450

[B40] Wu S. M. Liu J. H. Shu L. H. Chen C. H. (2015). Anti-oxidative responses of zebrafish (*Danio rerio*) gill, liver and brain tissues upon acute cold shock. Comp. Biochem. Physiol. A 187, 202–213. 10.1016/j.cbpa.2015.05.016 26025641

[B41] Wu Y. Feinstein S. I. Manevich Y. Chowdhury I. Pak J. H. Kazi A.. (2009). Mitogen-activated protein kinase-mediated phosphorylation of peroxiredoxin 6 regulates its phospholipase A(2) activity. Biochem. J. 419, 669–679. 10.1042/BJ20082061 19140803 PMC2770719

[B42] Yu S. Mu Y. Ao J. Chen X. (2009). Peroxiredoxin IV regulates pro-Inflammatory responses in large yellow croaker (*Pseudosciaena crocea*) and protects against bacterial challenge. J. Proteome Res. 9, 1424–1436. 10.1021/pr900961x 20099887

[B43] Zheng W. J. Hu Y. H. Zhang M. Sun L. (2010). Analysis of the expression and antioxidative property of a peroxiredoxin 6 from Scophthalmus maximus. Fish Shellfish Immunol. 29, 305–311. 10.1016/j.fsi.2010.04.008 20420920

