# Supplementary material for: The Antioxidant Peroxiredoxin 6 (Prdx6) Exhibits Different Profiles in the Livers of Seawater- and Fresh Water-Acclimated Milkfish, Chanos chanos, upon Hypothermal Challenge
Source: Front Physiol. 2016 Nov 29;7:580. doi: 10.3389/fphys.2016.00580 (PMC5126087; doi:10.3389/fphys.2016.00580)
Supplement: Supplementary file 1 [file Table1.DOCX]

**Supplementary Table S1. Primer sequences used in this study**

| **Gene** | **Primers** | **Sequence** | **Application** |
| --- | --- | --- | --- |
| *Ccgapdh* | Ccgapdh-qF824 | TCCTGCACCACCAACTGC | qPCR |
|  | Ccgapdh-qR1002 | GAGGCAGGGATGATGTTCTG |  |
| *Ccprdx6* | Ccprdx6-qF503 | CTGTACCCCGCCACCAC | qPCR |
|  | Ccprdx6-qR620 | CTTTATCACCTGGCTTCCAGTC |  |
|  | Ccprdx6-1F | GGCCCGCAGAATCTCGTTAAAGTTGC | RACE-PCR |
|  | Ccprdx6-1R | CTTTATCACCTGGCTTCCAGTCCACA |  |
